# A Microscopic “Social Norm” Model to Obtain Realistic Macroscopic Velocity and Density Pedestrian Distributions

**DOI:** 10.1371/journal.pone.0050720

**Published:** 2012-12-05

**Authors:** Francesco Zanlungo, Tetsushi Ikeda, Takayuki Kanda

**Affiliations:** 1 Intelligent Robotics and Communication Laboratories, ATR, Kyoto, Japan; 2 CREST, Japan Science and Technology Agency, Tokyo, Japan; University of Maribor, Slovenia

## Abstract

We propose a way to introduce in microscopic pedestrian models a “social norm” in collision avoiding and overtaking, i.e. the tendency, shared by pedestrians belonging to the same culture, to avoid collisions and perform overtaking in a preferred direction. The “social norm” is implemented, regardless of the specific collision avoiding model, as a rotation in the perceived velocity vector of the opponent at the moment of computation of the collision avoiding strategy, and justified as an expectation that the opponent will follow the same “social norm” (for example a tendency to avoid on the left and overtake on the right, as proposed in this work for Japanese pedestrians). By comparing with real world data, we show that the introduction of this norm allows for a better reproduction of macroscopic pedestrian density and velocity patterns.

## Introduction

In this work we tackle the problem of describing the behaviour of pedestrians in real world environments using a microscopic (i.e., based on individual pedestrian motion) model that takes into account the asymmetrical behaviour that pedestrians exhibit due to the presence of (often implicit or subconscious) social norms. The separation of counter-flows in pedestrian motion has been long studied from both an experimental and simulation point of view [1]–[3]. Most pedestrian collision avoiding models can reproduce the counter-flow separation in a corridor, but they usually do it in a symmetrical way, i.e. the flows may be generated both on the right or left side of the corridor. It has nevertheless been reported [4] that in most countries the separation of flows follows a “non-written rule”, i.e. the separation of the flows almost always happens on the same side (the side being dependent on the cultural norm, for example flows are reported to be on the right side in continental Europe, and on the left side in Japan). This norm may be represented as a (un)conscious choice to walk on a given side of the corridor (i.e., as a modification of the path choice mechanism of the pedestrian) but it has also been suggested that it may due to a bias in the collision avoiding behaviour of pedestrians [5]. According to this approach, the “social norm” that makes pedestrians walk on a given side of a corridor is still “emergent”, i.e. it originates from multiple pedestrian interactions, and can be simulated without major modifications in the modelling of the collision avoiding mechanism. Nevertheless, the presence of this bias (*microscopic social norm*) in collision avoiding has nontrivial effects on the macroscopic flow separation, not only affecting the direction in which the separation occurs but also enhancing the velocity and stability with which the flow divides.

In this paper we extend the previous research on the subject by accounting for the evidence, that we report in this work, of a social norm not only in collision avoiding but also in overtaking behaviour. In doing that we also provide a realisation of the behavioural bias that can be trivially applied to any collision avoiding model and at the same time is grounded on the concept of (microscopic) “social norm”, i.e. on the (possibly unconscious) expectation that also the interaction partner will adopt the same norm in avoiding and overtaking. After introducing such a bias in two different collision avoiding models, we investigate to which extent its introduction allows for a better reproduction of the density and velocity patterns observed in real world environments.

We believe that the presence of such social norms affects the self-organisation behaviour of pedestrians in counter-flows and in a single flow in a corridor (an aspect that is overlooked if the overtaking norm is not considered), and we believe that the introduction of the correct behavioural norm in pedestrian models may improve our ability to simulate and predict the behaviour of pedestrians also in more complex and realistic environments.

## Data Collection

We collected pedestrian trajectory data in an underground pedestrian facility in Umeda (downtown Osaka), Japan, in a location connecting a shopping area with a railway station. This location was chosen due to the absence of shops and other facilities (so that the pedestrians are expected to exhibit pure “goal-oriented” behaviour, i.e. they use the corridor just as a connection between an origin and a goal both located outside the corridor), and presents an average pedestrian density that allows for a good automatic tracking of pedestrian trajectories with our laser sensor technology [6]. The pedestrian density range that this location exhibits, corresponding to the normal condition of a shopping mall or average size station outside rush hour time, is quite low with respect to the usual range of interest in pedestrian studies, but it is high enough to present the macroscopic effects of the “social norm” and we believe that the insight obtained about pedestrian behaviour at these densities can be useful in the analysis and simulation of higher density behaviour. A detailed description of the experimental location and the data collection may be found in [7] and in the Materials and Methods section (The data set is available at https://sites.google.com/site/francescozanlungo/pedestriandata).

We spotted in the data collection location three “ideal corridors”: corridor 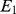, of width 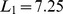 meters, corridor 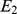, of width 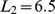 meters, and corridor 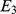 (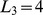 meters). Our definition of “ideal corridor” corresponds to the presence of straight walls, constant width, absence of shops, density and velocity patterns symmetrical along the corridor’s axis, and exclusive “goal-oriented behaviour” in pedestrians (moving along the corridor without pursuing other activities). As we have shown in [7] and discuss in Materials and Methods, 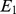 and 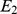 approximate to a very good extent the “ideal” behaviour, while 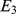 does it to a lower degree, but we decided to include it in our analysis since it provides information about a narrower environment.

**Figure 1:**
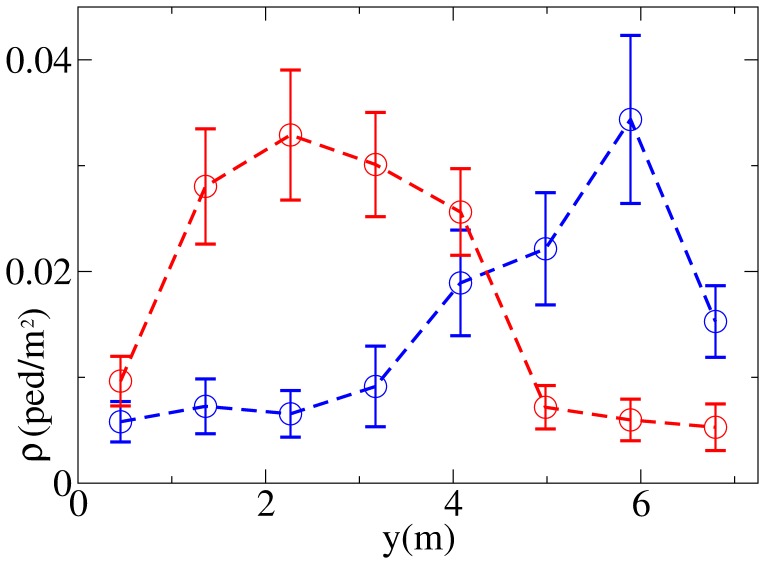
Density distributions as measured in the environment 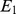 (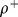 in red, 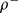 in blue, axes chosen in such a way that walking on the left corresponds to have 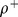 on the left of the figure). Error bars are obtained as standard deviations of values of 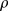 averaged over time windows of length 1200 s.

**Figure 2:**
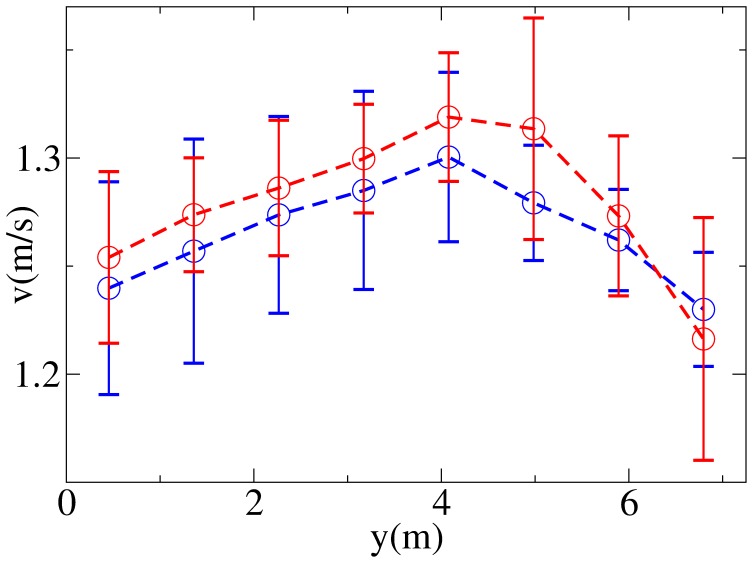
Velocity distributions as measured in the environment 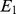 (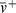 in red, 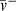 in blue). Error bars are obtained as standard deviations of values of 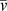 averaged over time windows of length 1200 s.

We choose for each environment a Cartesian reference frame with the 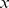 axis along the corridor’s axis (so that the 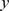 coordinate represents the distance from one of the walls) and divide pedestrians in two groups according to the value of their 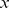 velocity component (i.e. their walking direction): the group of pedestrians with positive velocity 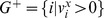 (where 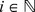 is the pedestrian label) and that of pedestrians with negative velocity, 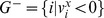. We divide each corridor in 8 “lanes” of width 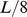 and compute in each lane 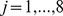 the average density 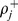 of pedestrians in group 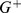, along with 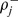 for pedestrians in 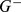, as well as the corresponding average scalar velocities (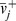 and 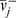; the notation 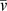 is used to distinguish the macroscopic average from individual scalar velocities 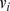). Figs. 1 and 2 show the resulting patterns in environment 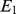. In order to obtain these figures we average over the whole observation time (20400 seconds in 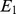 and 21600 seconds in 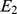 and 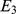), and over time windows of 1200 seconds (long enough to define macroscopic quantities such as 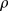 and 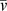 but short enough to study their time variation and stability) that are used to obtain the standard deviation error bars. Fig. 1 shows that while the average density in each group 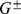 changes moderately with time, the density pattern in each flow is quite stable, and pedestrians exhibit a strong tendency to walk on the left side of the corridor (right of the figure for pedestrians in 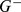). Fig. 2 shows that while the velocity exhibits in general a large variation on the right side of the corridor (left of the figure for pedestrians in 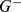), where a reduced number of pedestrians walk and thus fluctuations are stronger, there is a more clear pattern on the left side, and in particular a tendency to walk with a lower velocity when close to the wall, and a higher one when close to the centre of the corridor.

## Bias (Microscopic Social Norm)

### Experimental Evidence

As discussed at the end of the previous section, even at these relatively low values of average density (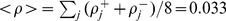 pedestrians per square meter in 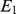, 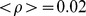 in 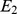 and 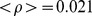 in 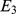) we observe in each environment a clear separation of flows (*macroscopic social norm*), always in agreement with the Japanese convention (walking on the left side of the corridor). We also observe a tendency to walk with higher velocity in the centre of the corridor (regardless of the walking direction). While in our previous work [7] we investigated the possibility that these patterns are the result of the individual (i.e., independent of the interaction with the others) decision of the pedestrian, in this work we will follow the approach of [5] in which they observed a bias in collision avoiding behaviour and used it to model the asymmetry in the flow separation (asymmetry in the 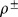 patterns) as an emergent property of the many pedestrian system. The analysis of the velocity patterns (maximum velocity in the centre of corridor) suggests us that pedestrians may follow a social norm also when overtaking (namely overtaking at the centre of the corridor, or, in the specific case of Japanese pedestrians, overtaking on the right, i.e. adopting as a norm the same rule used in vehicular traffic).

**Figure 3:**
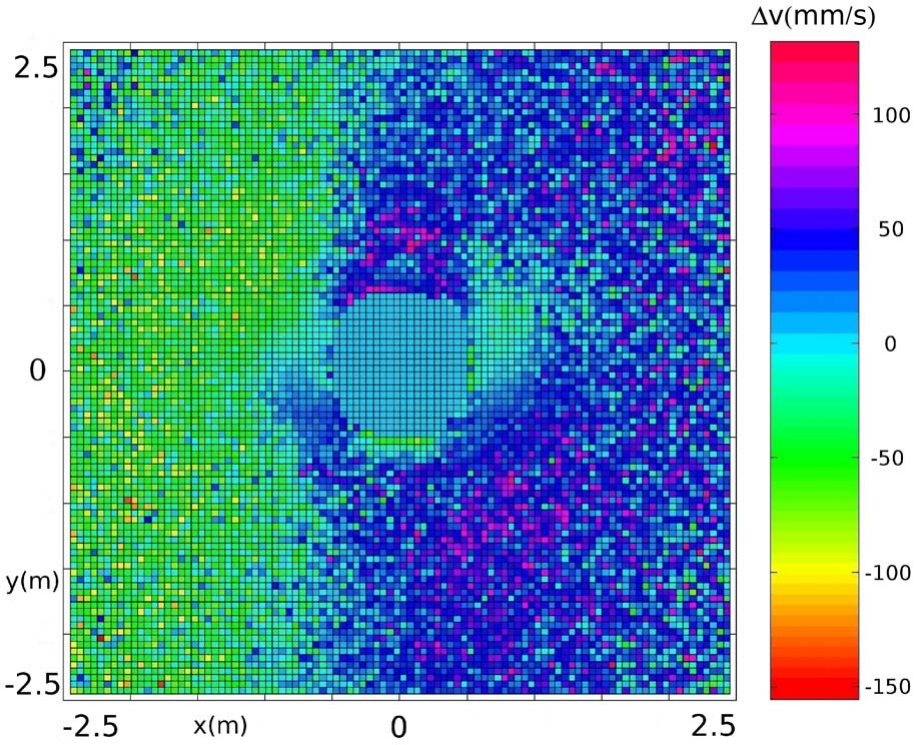
Relative velocity field around a pedestrian (based on all available observational data in the data collection location). The frame is centred on pedestrian 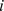, the 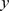 axis gives the relative position of pedestrian 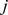 in the direction of 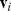 (i.e., 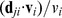), while the 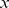 axis gives the relative position from left to right. The colour-bar reports average 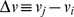 values in mm/s. The difference between values on the left and right, along with the very high relative velocity (violet) area on the back-right, suggest the presence of a right-oriented overtaking norm. The low velocity on the back (high velocity on the front) is probably related to collision avoiding behaviour for pedestrians moving in the same direction, while the low velocity area on the right and high velocity area on the left are probably related to group behaviour (friends that find themselves ahead will slow down and vice versa).

To better investigate the presence of such a norm, we measure the relative velocity between nearby pedestrians for all pedestrians in the data collection location. For each pedestrian 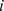 we define a Cartesian frame centred in the pedestrians’ position and with the 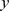 axis aligned with their velocity. Then we divide the space in square cells of linear size 0.05 meters and for each cell we measure and average the velocity difference 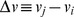 of 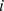 with respect to each pedestrian 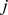 located in the cell, under the condition 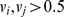 m/s and 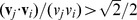 (empirical thresholds for “goal-oriented” pedestrians moving in the same direction). Fig. 3 reports a clear tendency for positive values on the right side, and negative values on the left, suggesting the presence of the proposed overtaking norm.

In the following we are going to introduce two different models (or *conditions*, to discriminate from collision avoiding model), one describing only the collision avoiding norm, the other describing both collision avoiding and overtaking norms.

### Position Bias (TP) or (only) Collision Avoiding Norm

**Figure 4:**
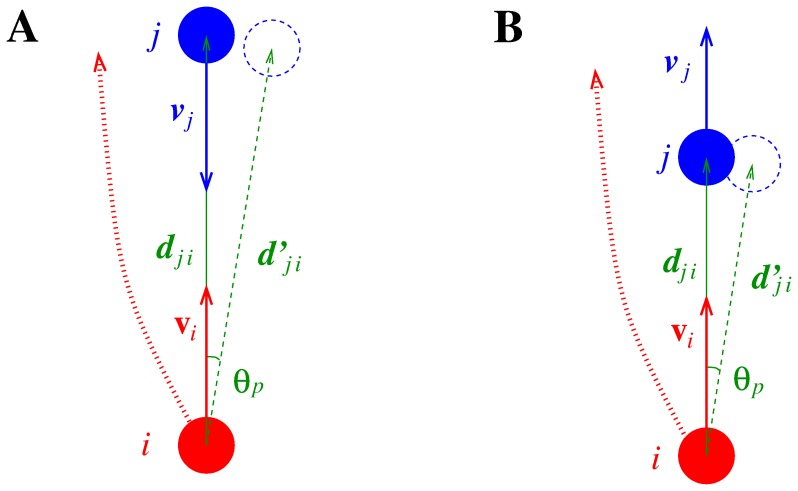
Collision avoiding and overtaking in pedestrian models using the TP (Tilt in Position) condition.

We first introduce a model that can describe the proper collision avoiding norm but fails to describe the overtaking one. Since basically any (continuous space) collision avoiding model determines the collision avoiding strategy of a pedestrian with respect to an opponent on the basis of the position and velocity of the deciding and the opposing pedestrian, a way to introduce a bias to explain the (Japanese) tendency to avoid on the left is (simplifying the approach proposed in [5] in such a way that can be implemented in any collision model) to rotate the relative distance vector from pedestrian 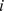 to pedestrian 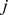, i.e. 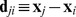, of a clockwise angle 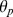 as
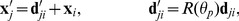
(1)and use it in the computation of the collision avoiding strategy of 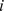 (see Fig. 4 **A**; the continental Europe norm is obviously obtained using a counter-clockwise angle). We nevertheless believe that this bias, which we name TP (Tilt in Position) condition, has a conceptual and a practical shortcoming. The conceptual one is that, although it provides an empirical rule to obtain the desired collision avoiding behaviour, it does not seem to provide any grounding to explain the pedestrian behaviour, i.e. the rotation in the relative position of the opponent seems just a computational trick to obtain the correct norm, but is not related to nor proposes any explanation of the pedestrian’s cognitive process when applying the norm. The practical one is that using this bias induces a tendency to avoid on the left, and overtake on the left (i.e. close to the walls), as shown in Fig. 4 **B**.

### Velocity Bias (TV) or Collision Avoiding and Overtaking Norm

**Figure 5:**
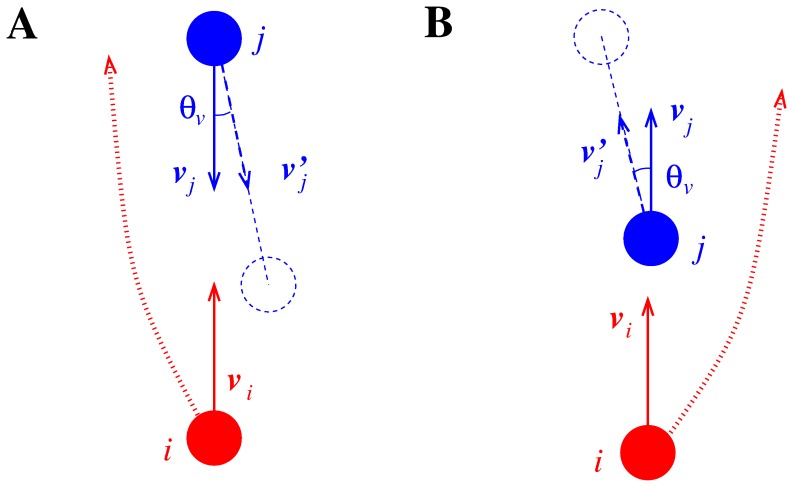
Collision avoiding and overtaking in pedestrian models using the TV (Tilt in Velocity) condition.

**Figure 6:**
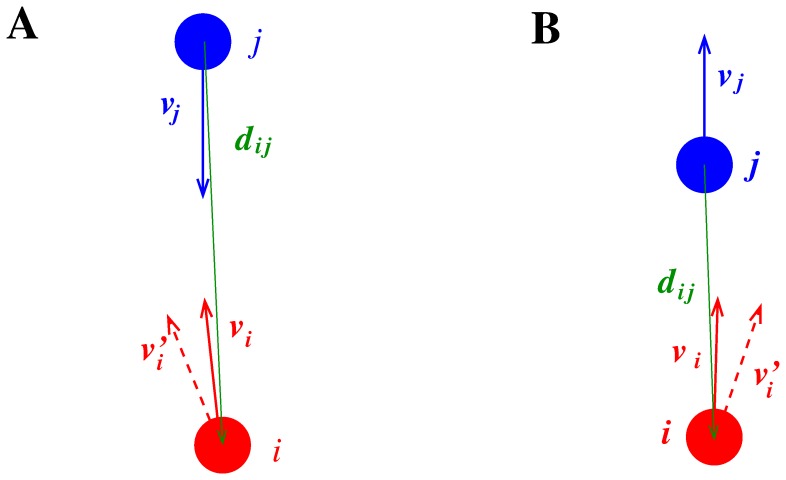
Difference between avoiding a collision and being overtook in pedestrian models using the TV (Tilt in Velocity) condition. A: since 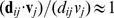, i.e. 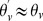, 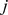 expects 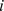 to avoid on the left. B: since 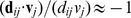, i.e. 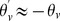, 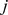 expects 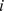 to overtake on the right.

We thus suggest a different bias, namely rotating the opponent’s velocity vector of a counter-clockwise angle 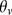, so that in collision avoiding, by predicting the future motion of 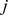 as directed on her right, 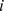 will deviate on the left, but when performing overtaking she will deviate on the right by expecting 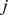 to deviate on the left (see Fig. 5). Not only this bias explains correctly both the expected avoiding and overtaking norms, but it can be considered as an actual realisation of the (microscopic) norm, since it can be justified from a conceptual point of view as the expectation that the opponents will modify their velocity according to the same norm (avoiding on the left, and moving on the left when overtaken to give space to the overtaker). To better describe also the expectation of the overtaken to be passed on the right, we define our TV (Tilt in Velocity) bias as a rotation of the opponent’s velocity.
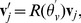
(2)where
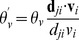
(3)(see Fig. 6). Such a modification accounts also for the reduction of the effect of the bias in “crossing encounters”, i.e. when 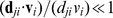 (since no clear social norm is defined in such a situation), and thus allows for applications to environments more complex than “ideal corridors”. We notice that while the TP bias can be applied also to models that, as the Circular Specification of the Social Force Model (SFM) [8], do not use the opponent’s velocity in the determination of the avoiding strategy (while the TV bias would be ineffective in those models), it can be shown that using the opponent’s velocity is necessary to properly describe pedestrian motion, in particular outside the high density regime [9]–[11].

## Simulations

In this section we try to reproduce the 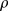 and 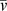 distributions observed in our data collection campaign by using purely collision avoiding pedestrian models, comparing the performance of the different bias conditions. The observed 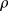 and 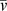 patterns are stable in time and along the whole data collection location, i.e. on the time scale of tens of seconds and meters over which we could follow individual pedestrians we did not observe the process of formation of these patterns. We thus suggest that if the patterns are the results of multiple pedestrian interactions, the space scale for their formation is that of the larger Umeda pedestrian area, i.e. hundreds of meters or even a few kilometres. To reproduce these patterns we thus perform simulations with ideal corridors of widths and average densities corresponding to those of 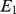, 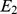 and 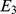, but we use longer lengths and periodic boundary conditions in order to have pedestrians walking in the environment for time scales of hours and distances of kilometres. The observed patterns are compared to the simulation ones, and the parameters of the models that better reproduce the data are optimised through a Genetic Algorithm (GA), which is a very valuable method for optimising the output of a complex model using a simple fitness function [12], and has been used with success in optimisation of pedestrian models [9]–[11], [13], [14]. The final fitness (similarity score) of the best solution is used as an evaluation function for the ability of a given model to reproduce the observed patterns (see the Materials and Methods section for details).

Both suggested biases can be straightforwardly applied to any collision avoiding pedestrian model using position and velocity information. In our analysis we use the Elliptical Specification II of the SFM (ES) [9] and the Collision Prediction Specification (CP) [9], two models that are quite different in their formulation but yield very similar results (see Materials and Methods for details). In determining the 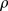 and 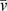 distributions in a corridor, also the interaction with the walls has an important role. In ES we implement the interaction with walls using forces whose intensity decreases exponentially with the distance from the walls, as it is usually done in the SFM framework (i.e. the interaction with the walls is velocity-independent and can influence the 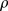 but not the 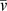 distribution), while in CP the possible collisions with the walls are explicitly computed, introducing a velocity dependence in the interaction with the walls (since faster pedestrians may collide earlier and faster with walls, the resulting force leads them to walk farther from the walls *regardless of the overtaking behaviour*).

We evaluate the similarity between the simulated and observed distributions using the following fitness function, that tests the ability of simulations to reproduce the experimentally observed 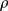 and 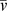 distributions by measuring the ratio of the difference between simulated and observed patterns over the range of values assumed by the observed distribution (this fitness function was chosen to evaluate properly both 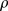 and 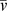 distributions, and to reduce the effect of fluctuations; see Materials and Methods for a detailed justification). In detail, let us name 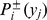 and 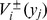 the simulated 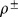 and 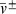 distributions in environment 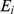, for each flow, evaluated in the centre of each 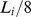 wide lane (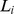 is the width of environment 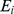),
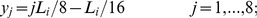
and 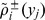, 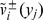 the corresponding observed distributions, normalised in such a way that, defining
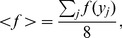
(4)we have



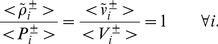
(5)Let us also denote the range of values assumed in each distribution as.
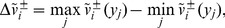
(6)

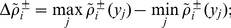
(7)the fitness function is defined as
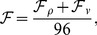
(8)where
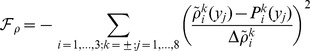
(9)and



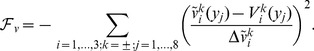
(10)This function averages the square of the error relative to the range of values assumed in the distribution for all the 96 evaluation points (8 points in 4 distributions for 3 environments).

Simulations are performed using the CP and ES models with the TP, TV biases and without bias (T0). Given the stochastic nature of the fitness function (due to the stochasticity in the pedestrian velocity distribution, relative weight of flows and noise in the model output, see also Materials and Methods), and the possibility that single runs of GA are trapped around local maxima for a time comparable to the overall iteration number, we perform 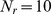 independent GA runs for each model and condition. For each independent GA run we record the fitness value of the best found solution, and the average value and standard deviation of best solutions’ absolute fitness value over the 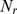 runs are used as an evaluation function of the model and condition (evaluation function 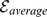; since the absolute value of fitness gives the difference between observed and simulated distributions, a lower 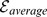 accounts for a better performance). At the same time it is important to record the parameter set of the overall best solution (the best solution with the maximum fitness function over the 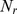 runs), whose performance is then statistically evaluated over 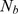 independent tests (evaluation function 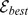). We may say that 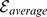 provides information about the ability of the GA to find a good solution for a given model and condition, and the stability of this solution, while 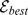 provides information about the best possible performance (global maximum) of the model (see Materials and Methods for more details on evaluation functions).

## Results

Table 1 shows the average value and standard deviation over different GA runs for all models and conditions (evaluation function 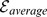), while Table 2 shows the performance of best solutions (evaluation function 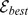). For each model we have a difference of order 3–4 standard deviations (in 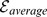, the difference is much larger in 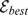) between the TP and T0 conditions, and around 2 standard deviations (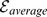) between TV and TP, showing the improvement due to the introduction of a collision avoiding norm, and the further improvement due to the introduction of the overtaking norm. It is interesting to notice the large difference (up to 6 standard deviations for TP and TV) between the CP and ES models. We do not believe that this difference is due to some pitfall in the description of collision avoiding in ES with respect to CP since the two models have similar performances in describing individual behaviour [11], nor that the proposed “social norms” (TP and TV) cannot be applied properly to ES. According to our interpretation, the better performance of CP is due to its ability to describe the tendency of faster pedestrians to walk in the centre of the corridor regardless of overtaking (due to its velocity-dependent wall interaction). If two different tendencies are present, i.e. walking preferentially closer to the centre of the corridor while walking fast, *and* overtaking on the right, a model like CP-TV, that can describe both tendencies, should outperform a model as ES-TV that can describe only the overtaking one. Since CP-TP outperforms ES-TV of 3 standard deviations, it appears that in our environments overtaking is not the leading factor. Nevertheless, since 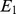 is the largest and most dense environment, it may be expected that in 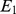 overtaking happens more often, and thus the overtaking norm would be relatively more important in describing the velocity distributions of that environment.

**Table 1:** Models and conditions performance (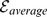).

	T0	TP	TV
ES	0.079±0.009	0.061±0.004	0.052±0.005
CP	0.059±0.005	0.037±0.004	0.032±0.002

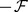
 for different models and conditions (evaluation function 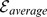, i.e. average performance and stability of the model). ES stands for the Elliptical Specification model, while CP stands for the Collision Prediction model. TP stands for the Tilt in Position condition, TV for the Tilt in Velocity one, while T0 for the absence of social norms. Averages and standard deviations are over 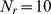 different GA runs.

**Table 2:** Models and conditions performance (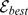).

	T0	TP	TV
ES	0.070±0.002	0.0593±0.0004	0.044±0.001
CP	0.064±0.004	0.037±0.001	0.031±0.001

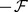
 for different models and conditions (evaluation function 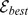, i.e. best performance of the model). ES stands for the Elliptical Specification model, while CP stands for the Collision Prediction model. TP stands for the Tilt in Position condition, TV for the Tilt in Velocity one, while T0 for the absence of social norms. Averages and standard deviations are obtained over 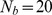 tests of the overall best solution over the 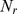 runs of Table 1.

We thus performed a second test calibrating only on the 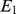 velocity and density distributions, obtaining the results of Tables 3 and 4, which are in agreement with our hypothesis by showing that in the description of the 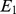 environment overtaking is more important than the tendency to walk closer to the centre while walking faster (CP-TP is outperformed by ES-TV of one standard deviation and by CP-TV of two standard deviations in 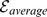).

**Table 3:** Models and conditions performance on 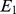 (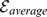).

	TP	TV
ES	0.055±0.007	0.033±0.005
CP	0.040±0.007	0.029±0.005

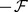
 for different models and conditions after calibration only on the 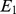 environment (evaluation function 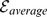, i.e. average performance and stability of the model). ES stands for the Elliptical Specification model, while CP stands for the Collision Prediction model. TP stands for the Tilt in Position condition, while TV stands for the Tilt in Velocity one. Averages and standard deviations are over 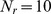 different GA runs.

**Table 4:** Models and conditions performance on 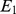 (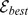).

	TP	TV
ES	0.045±0.001	0.031±0.001
CP	0.037±0.001	0.025±0.001

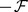
 for different models and conditions after calibration only on the 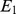 environment (evaluation function 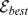, i.e. best performance of the model). ES stands for the Elliptical Specification model, while CP stands for the Collision Prediction model. TP stands for the Tilt in Position condition, while TV stands for the Tilt in Velocity one. Averages and standard deviations are obtained over 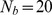 tests of the overall best solution over the 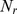 runs of Table 3.

**Figure 7:**
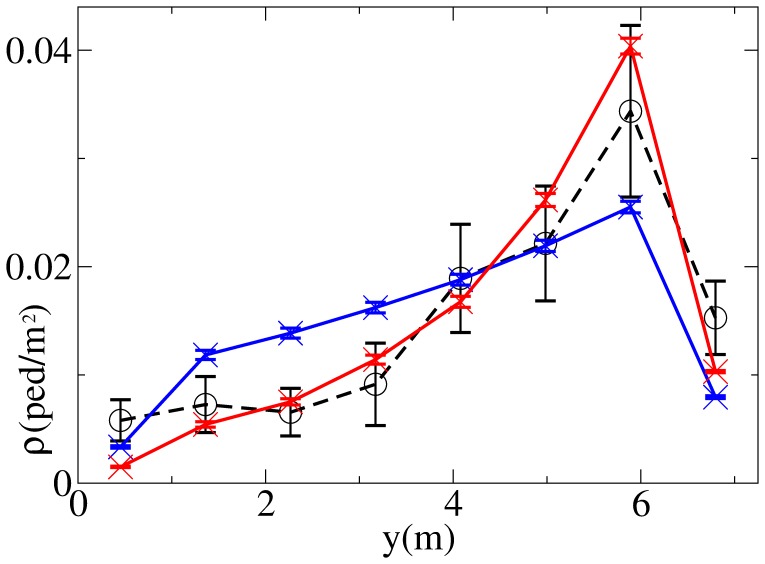
Comparison between the observed density distribution 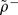 (black) and simulated density distributions 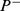 (blue and red) in 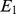. Both simulated distributions are obtained using the ES (Elliptical Specification) model, the blue line showing results obtained under the TP (Tilt in Position) condition, the red line results obtained under the TV (Tilt in Velocity) condition. Average values and standard deviations (error bars) for simulated distributions are obtained over 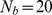 tests (using 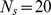) of the overall best solution over the 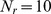 independent runs of the GA (calibration over all the environments). Observed data error bars are obtained as in Fig. 1.

**Figure 8:**
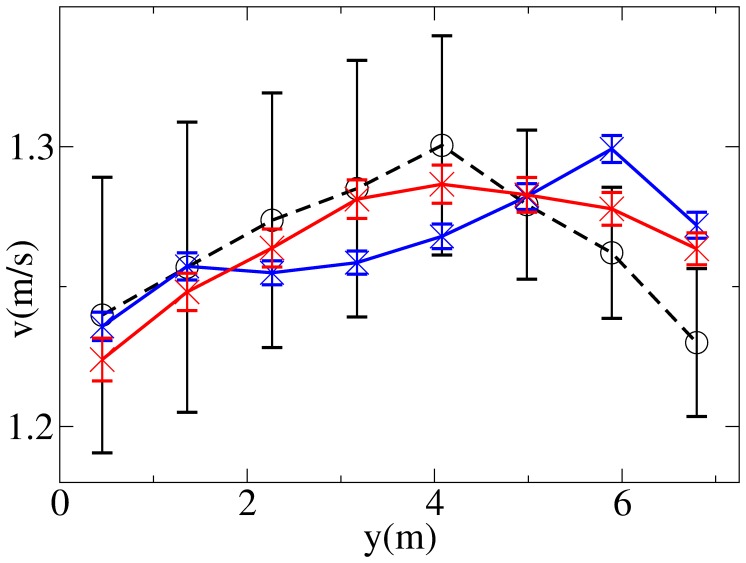
Comparison between the observed velocity distribution 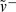 (black) and simulated velocity distributions 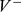 (blue and red) in 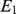. Both simulated distributions are obtained using the ES (Elliptical Specification) model, the blue line showing results obtained under the TP (Tilt in Position) condition, the red line results obtained under the TV (Tilt in Velocity) condition. Average values and standard deviations (error bars) for simulated distributions are obtained over 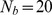 tests (using 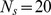) of the overall best solution over the 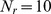 independent runs of the GA (calibration over all the environments). Observed data error bars are obtained as in Fig. 2.

Figs. 7, 8 show the comparison between the results obtained using the ES model under the TV and TP conditions on the 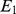 environment, after calibration over all environments. Since the fitness function is quadratic, it penalises large errors. As a result, in order not to choose parameter values that would describe a 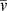 distribution very different from the experimental one, the GA chooses for the ES-TP method values of parameters that generate a weak separation between flows, since a stronger flow separation would require a stronger microscopic “social norm” and thus, in the TP condition, high velocities close to the wall (see also the Parameter values section in Materials and Methods).

### Conclusions

In this work we provided experimental evidence about the tendency of Japanese pedestrians to walk preferentially on the left side of corridors, to walk with higher velocity when close to the centre of the corridor, and in general to overtake on the right. Based on these observations, in order to better describe the pedestrian behaviour, we suggested two different ways to implement “social norms” in any microscopic pedestrian model that uses velocity based information. The first (Tilt in Position) norm describes only the tendency of pedestrians to avoid collisions by deviating on the left, introducing a bias through a rotation in the opponent’s relative position; while the second (Tilt in Velocity) one describes both the tendency to avoid on the left and overtake on the right by rotating the opponent’s velocity vector (the Tilt in Velocity social norm can be justified from a cognitive point of view as the expectation that the opponent would follow the same strategy). We have shown, using two different collision avoidance models, that the introduction of such social norms allows for a better reproduction of observed pedestrian velocity and density patterns with respect to models not using any kind of social norm. Furthermore, we have shown that the social norm describing both collision avoidance and overtaking behaviours outperforms the norm that describes only the bias in collision avoiding. We also found that models including a tendency to walk closer to the centre of the corridor for fast walking pedestrians, *regardless of overtaking behaviour*, may describe better the velocity distribution of actual pedestrians, in particular at low densities. Nevertheless, models that include a description of this tendency *and* of the overtaking norm outperform models without overtaking norm, and the overtaking behaviour becomes dominant at higher densities. Assuming that these kinds of norm are present also at higher densities, a proper introduction in simulation methods should enhance the ability to simulate pedestrian flows and design pedestrian facilities able to sustain diverse pedestrian streams.

A relevant part of pedestrian crowds is composed of groups [15]–[17], as confirmed also by our analysis of relative velocities. The group behaviour affects the macroscopic behaviour of pedestrians [18] and thus also the density and velocity distributions, and the introduction of group behaviour in simulations, along with the development of the necessary “group-related social norms” represents an interesting development of the present work.

We also believe that a cross-cultural study would be extremely interesting, to compare qualitative and quantitative differences between the norms that we have observed in Japanese pedestrians and those occurring in other countries and cultures.

## Materials and Methods

### Data

Our data campaign is described in detail in [7] (the environments 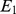, 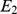 and 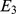 are named, respectively, 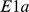, 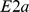 and 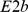 in that work). Our definition of an ideal corridor is based on a qualitative analysis of the environment (absence of shops, intersections, obstacles; straight walls) and a qualitative and quantitative analysis of the data (denoting the 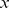 axis as the corridor’s axis, density should be almost invariant along 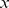, furthermore we require at least 90% of the data points recorded in the environment to satisfy our empirical definition of “goal-oriented behaviour”, i.e. 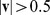 m/s, 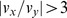). Density and velocity were initially computed on squares of linear size 25 cm using all data, but eventually only data satisfying the “goal-oriented behaviour” condition are used to obtain the distributions analysed in this paper. 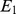 (length 23 m) and 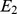 (10 m) satisfy all our conditions, while 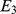 (17 m) actually crosses another corridor and only 60% of the data satisfies the “goal-oriented behaviour” condition.

### Collision Avoiding Models

The ES model is a SFM specification that takes into account also relative velocity information to better describe pedestrian motion [9]. The force on pedestrian 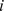 determined by pedestrian 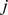 is given by
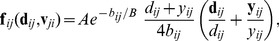
(11)where 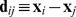 and 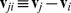 are, respectively, the relative distance and velocity between pedestrians 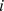 and 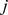,



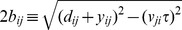
and



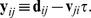


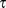
 was originally introduced as the time of a pedestrian stride, but we found [11] that a value of 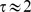 s better describes the prediction of other people’s motion that pedestrians perform at the densities of interest in this work. The interaction with the walls is implemented as a force orthogonal to the walls with magnitude



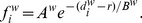
(12)Here 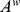 and 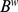 are wall specific parameters, 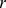 is the pedestrian size (radius) and 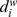 the current distance of pedestrian 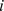 from the wall.

The CP model [11] introduces in the SFM framework concepts developed in the velocity-based models [19]–[21], using in the original equations of the Circular Specification [22], instead of the current distance between the pedestrians, the distance they will have at the moment of maximum approach. In detail, pedestrian 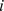 computes for each pedestrian 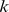 in the environment the time 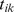 at which they will reach the minimum relative distance, assuming they will maintain their current velocities. 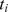 is defined as the minimum over 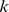 of 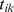. Then the force on 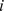 determined by a particular pedestrian 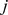 is computed as.
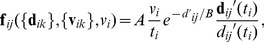
(13)where 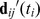 is the predicted relative distance at time 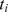 (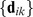 and 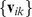 are the sets of relative distances and velocities with respect to all the pedestrians 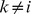 in the environment, which are necessary to determine 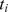). The interaction with walls is implemented by using predicted distances to walls in eq. 13, along with wall specific parameters 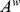 and 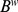 (collision times with walls are considered when computing 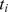). In this work we made the model more stable by applying the condition



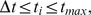
where 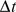 is the integration step and 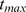 a new parameter of the model. The performance of the two models is very similar at the densities investigated in this paper [11].

### Simulations Settings

In all simulations we use a time step 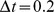 s. The physical dynamics of pedestrians is approximated as that of hard discs of radius 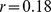 m (at the density of interest in this paper the physical interactions between pedestrians are negligible and this condition is just used to ensure non-overlapping in the rare occurrence of a collision). All the corridors in our simulation environments are 500 m long. In order to have the same overall density (i.e., 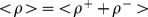) that we observed in 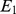, 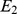 and 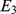, we place 120 pedestrians in a 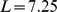 m environment, 65 in a 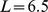 m one, and 42 in a 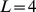 one. A test of a possible solution (set of parameters for the model and condition) consists of 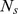 *simulations*. In each single simulation pedestrians are assigned to 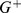 with probability 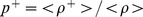 and to 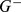 with probability 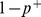, while their preferred velocities are randomly determined by a Gaussian distribution with mean 1.28 m/s and deviation 0.2 m/s, corresponding to the observed distribution of average velocities in the data collection location. Virtual pedestrians walk in the environment for 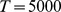 s. In order to reduce the effect of fluctuations and to obtain time-stable “asymptotic” distributions, the 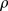 and 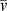 distributions used in the evaluation of the fitness function are obtained as the average over the last 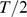 seconds of 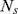 statistically independent simulations. In order to check the time stability of these distributions we perform also simulations of length 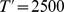 s without observing significant changes, i.e., performing the tests in Tables 2 and 4, we have differences between the 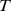 s and 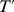 s tests smaller than the corresponding standard deviations. During the evaluation of solutions in the GA we use 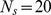 simulations for each fitness function evaluation, while during the test of best solutions (i.e., in the 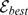 evaluation) we use 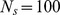. In our simulations all pedestrians use the same model and condition parameters, but in order to reproduce (at least from a purely statistical point of view) the unpredictability and diversity of human behaviour we add noise to the model output (the value of the noise intensity is one of the GA parameters).

### GA Settings and Parameters

The GA uses 30 genomes and 30 generations, tournament selection (two best solutions in two pools of 3 randomly picked ones are used for mating), crossover and random mutation with probability 0.03 (parameters are coded as floating point numbers and modified with Gaussian white noise; each parameter is constrained between a maximum and minimum value and the standard deviation of the Gaussian mutation is one tenth of the parameter range). The ranges of the model parameters are chosen in such a way that they do not differ strongly from those that describe the local behaviour of pedestrians as reported in [11].

The parameters of the ES model are: the noise in the velocity output of the model 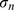 (standard deviation of normal white noise added to the 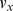 and 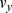 model output); the asymmetry parameter 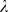 (see for example [11] for a definition); the inverse of the time scale to recover the preferred velocity, 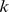; 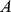; 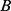; 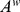; 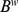; the range of interaction with pedestrians 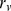 (pedestrians are ignored if their *current* distance is larger than 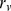); the interaction range with walls 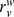; and 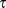.

The parameters of the CP model are: 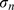; 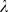; 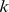; 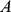; 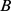; 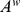; 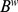; 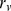 (pedestrians are ignored if their distance *at the moment of maximum approach* is larger than 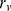); 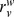; and 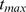.

The parameter ranges (including the “social norm” parameters 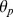 and 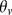) are reported in Table 5.

**Table 5:** Parameter ranges in models and conditions.

	ES	CP
*σ*_n_	0.2–0 m/s	0.2–0 m/s
*λ*	1–0	1–0
*k*	1.26–0.63 s^−1^	2.28–1.14 s^−1^
*A*	1.6–0.8 m/s^2^	2.26–1.13 m/s^2^
*B*	1.24–0.62 m	1.42–0.71 m
*A^w^*	1.6–0.1 m/s^2^	2.26–0.1 m/s^2^
*B^w^*	1.24–0.1 m	1.42–0.1 m
*r_v_*	10–3 m	10–0.5 m
*r_v_^w^*	3–0 m	3–0 m
*τ*	2.6–1.3 s	
*t_max_*		10–2 s
*θ_p_*	0.4–0 rad	0.4–0 rad
*θ_v_*	0.4–0 rad	0.4–0 rad

Ranges of values that the simulation parameters can assume, i.e. the GA chooses the parameters that better describe the measured data within these ranges. The values were chosen on the basis of the calibration on individual pedestrian trajectories performed in [11], in order to ensure that the microscopic behaviour of simulated pedestrians would not differ too much from that of real pedestrians. ES stands for the Elliptical Specification model, while CP stands for the Collision Prediction model.

### Fitness Function

Our work compares the ability to reproduce observed and simulated 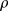 and 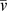 patterns. The major problem that we faced was to introduce a quantitative fitness function that could reflect the ability of the simulations to reproduce the “qualitatively salient” features of two distributions that are quite different between them. Both distributions may oscillate in their average value, since the number of pedestrians in each flow and the preferred velocity of pedestrians are chosen in a probabilistic way. These fluctuations can be reduced using a high number of simulation 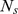 for each evaluation, as we do in the final tests on best solutions, but a high value of 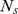 is extremely computationally expensive if used in the GA. Furthermore, the average value of the 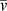 distribution is determined by the input of the preferred velocity distribution, but preferred velocities and average velocities are not the same, and their relation changes according to models, conditions and parameter values. For this reason we decided to scale the distributions in such a way that simulated and observed ones have always the same average value (eq. (5)). Since the two distributions are dimensionally different, it is necessary to use an adimensional quantity in the fitness function. The most straightforward solution would be the relative error, but while such a quantity can reach values around 1 for the 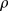 distribution, whose range goes from 0 to a maximum 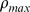, on the opposite, the relative error values assumed in the 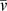 distribution are limited by the input of the preferred velocity distribution, i.e. a Gaussian centred in 1.28 m/s with a variance 0.2 m/s, and thus typically limited to a 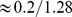 range. In order to give the same weight to the two distributions, we divide the absolute error by the ranges (eqs. (6–10)) obtaining relative errors (possibly) up to 1 both for 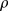 and 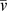.

### Evaluation Functions

For complex multi-dimensional parameter space problems as those faced in this work, a single run of the genetic algorithm may get trapped around a local maximum for a time comparable to the overall generation number. In order to avoid this problem we use 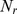 statistically independent GA runs to explore an as large as possible portion of the parameter space. By computing the average and standard deviation of the absolute value of the fitness of the best solution in each run (evaluation function 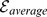), we obtain information about the average performance and stability of the GA calibration for each model and condition, which is reasonably related to the ability of the model and condition to reproduce the observed data. The best solution over the 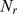 runs is obviously our best estimate for the global maximum of the problem, nevertheless given the stochastic nature of the problem, its value over a single test may not be significant. For this reason we also run 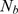 independent tests, each one composed by 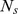 independent simulations (evaluation function 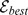), of this overall best solution, in order to obtain a good approximation of the value of the global maximum of the problem, i.e. of the performance of the model and condition. Note that while in general 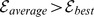, this relation might not hold for models and conditions strongly affected by fluctuations.

### Parameter Values

Tables 6 and 7 report the values of parameters after calibration in all models and conditions (average and standard deviation over the 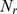 GA runs). We first notice that the value of 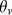 (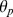) depends on the model, and specifically is lower for the CP model. This could be due to the fact that CP, by performing an explicit prediction of collision times, is more sensible to the tilts in velocity and position. We also notice that in general the value assumed by 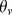 is larger than the value assumed by 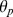; this could be due to the fact that, as we discussed in the Results section, the GA usually leads to a weaker microscopic social norm for TP, to avoid having high velocities close to the wall and thus a 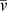 distribution very different from the experimental one. This hypothesis is also backed by the observation that in general 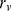 assumes a much higher value under TV than under TP: by reducing the interaction, TP manages to have a 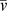 distribution as similar as possible to the observed one (Fig. 8), to the expenses of a weaker flow separation (Fig. 7).

**Table 6:** Parameter values after calibration in the ES (Elliptical Specification) model.

	ES-TP	ES-TV
*σ*_n_	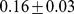 m/s	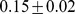 m/s
*λ*	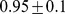	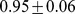
*k*	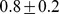 s^−1^	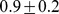 s^−1^
*A*	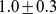 m/s^2^	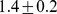 m/s^2^
*B*	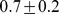 m	 m
*A^w^*	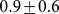 m/s^2^	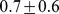 m/s^2^
*B^w^*	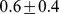 m	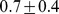 m
*r_v_*	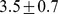 m	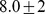 m
*r_v_^w^*	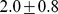 m	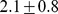 m
*τ*	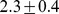 s	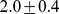 s
*θ_p_*	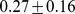 rad	
*θ_v_*		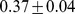 rad

TP stands for the Tilt in Position condition, while TV stands for the Tilt in Velocity one.

**Table 7:** Parameter values after calibration in the CP (Collision Prediction) model.

	CP-TP	CP-TV
*σ*_n_	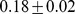 m/s	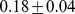 m/s
*λ*	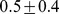	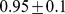
*k*	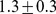 s^−1^	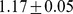 s^−1^
*A*	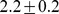 m/s^2^	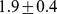 m/s^2^
*B*	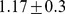 m	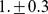 m
*A^w^*	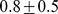 m/s^2^	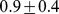 m/s^2^
*B^w^*	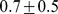 m	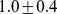 m
*r_v_*	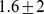 m	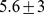 m
*r_v_^w^*	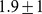 m	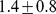 m
*t_max_*	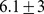 s	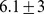 s
*θ_p_*	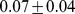 rad	
*θ_v_*		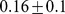 rad

TP stands for the Tilt in Position condition, while TV stands for the Tilt in Velocity one.

### Ethics Statement

No ethics statement is required for this work. Position recordings of pedestrians were made in public areas and the data were analysed anonymously.
